# Ridesourcing platforms thrive on socio-economic inequality

**DOI:** 10.1038/s41598-024-57540-x

**Published:** 2024-03-28

**Authors:** Arjan de Ruijter, Oded Cats, Hans van Lint

**Affiliations:** Department of Transport & Planning, Delft University of Technology, Delft, 2628 CN The Netherlands

**Keywords:** Socioeconomic scenarios, Computational science

## Abstract

Limited available market share data seems to suggest that ridesourcing platforms benefit from, even thrive on, socio-economic inequality. We suspect that this is associated with high levels of socio-economic inequality allowing for cheap labour as well as increasing the share of travellers with a considerably above-average willingness to pay for travel time savings and comfort. We test the relation between inequality and system performance by means of an agent-based simulation model representing within-day and day-to-day supply-demand interaction in the ridesourcing market. The model captures travellers’ mode choice with a heterogeneous perception of relevant time components, as well as job seekers’ participation choice with heterogeneous reservation wage. Our experiments cover scenarios for the entire spectrum ranging from perfect equality to extreme inequality. For several of such scenarios, we explore alternative platform pricing strategies. Our analysis shows a strong, positive relationship between socio-economic inequality and ridesourcing market share. This is the outcome of the combination of cheap labour and time-sensitive ridesourcing users, reinforced by network effects inherent to ridesourcing markets. We find that driver earnings are minimal in urban areas with large socio-economic inequality. In such contexts, drivers are likely to face a high platform commission, and yet, fierce competition for passengers.

## Introduction

In the gig economy, independent contractors provide services to consumers via intermediary platforms. Industries in which the gig business model has become especially prevalent include passenger transportation, delivery of food and goods, provision of household tasks, and care work^1^. The main benefit associated with working in the gig economy is autonomy^2–7^. In principle, gig workers can freely decide which platforms to work for, when to work for these platforms, which jobs to take, and how to fulfil these jobs. In reality, autonomy may be limited by rules and mechanisms imposed by platforms, as well as by demand fluctuations^7–11^. In fact, courts in the United Kingdom and the Netherlands have ruled that workers of ride-hailing provider Uber are self-employed only on paper^12,13^, assessing the relationship between drivers and platform to meet all conditions of traditional employment. In Amsterdam, there was a similar ruling for food delivery platform Deliveroo^14^, which led to its departure from the Netherlands^15^.

While average hourly earnings on gig platforms are not necessarily lower than the wage paid by employers that are active in similar industries^16^, gig workers lack access to social security provisions, including a guaranteed minimum wage, retirement plans and paid (sick) leave. Consequently, workers in the gig economy are exposed to much more uncertainty when it comes to income than employees. Essentially, gig workers now bear the risks that were previously carried by business and state. These risks include shrinking demand, as in the recent COVID-19 pandemic^17^, health issues, damage to assets and (financial) impropriety by customers^10,11^, but also chance in platform matching^18,19^. With a substantial share of gig workers relying on highly volatile income from platform work to cover costs of living^17,20^, the emergence of the gig economy may result in larger income inequality in society. More so, dependency on platform earnings limits gig workers’ freedom, which can contribute to below average earnings in the gig economy^21^.

While the provision of some services exchanged in the gig economy demands extensive training (e.g. writing, consulting, designing), many other gig economy services require limited skills. This includes driving, housekeeping and specific online tasks such as filling out surveys. As a result, many gig markets predominantly attract workers with a below average income^6,20^, in particular migrant workers^4,6,22,23^. Due to a scarcity of alternative income opportunities, these individuals might find themselves compelled to work for such platforms despite earning limited wages. While minimum wages ensure a foundational income for workers in conventional markets, self-employed individuals engaged in the gig economy do not have this income safeguard. This situation suggests that platforms within the gig economy are particularly likely to - more than service providers with employed staff - gain advantages from socio-economic disparities by leveraging inexpensive labour.

The ridesourcing (or ride-hailing) sector, represented by platforms such as Uber, Lyft, and DiDi, stands out as a gig industry that may significantly benefit from socio-economic disparities. Ridesourcing platforms not only profit from accessing abundant inexpensive labour but also cater primarily to individuals with above-average socio-economic positions. That is to say, users are assured a direct and private ride resembling a taxi service, at a typically substantially higher fare compared to traditional public transportation^24^. Moreover, as the average distance between a matched traveller and driver (at the time of assignment) decreases with the (double-sided) size of the ridesourcing market^25^, socio-economic inequalities may allow ridesourcing platforms to exploit network effects in matching that yield benefits for travellers (less waiting), drivers (less idle time) and platform (more profit through induced demand). Because taxi market supply is typically regulated, it is expected that taxi operators profit less from such network effects than ridesourcing platforms do.

**Figure 1:**
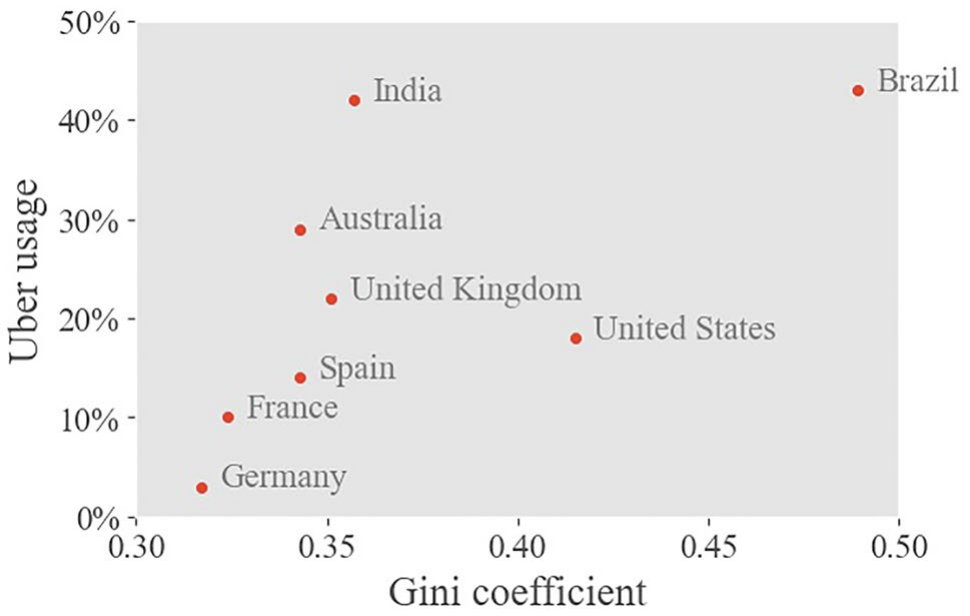
The share of adults in selected countries (based on data availability) that used Uber at least once in a 1-year period following April 1st 2021^26^ plotted against the country’s Gini coefficient for income inequality^27^. It suggests that a positive relationship exists between socio-economic inequality and ridesourcing market share.

Figure 1 seems to suggest that there may be a correlation between socio-economic inequality in a country and the usage of ridesourcing. This is based on data for just eight countries however, for which one-time Uber usage is used as a proxy for the prevalence of ridesourcing. Unfortunately, reliable city-level indicators for ridesourcing across numerous cities are not available. We therefore need alternative means to testify the relation between socio-economic inequalities and ridesourcing system performance. We argue that an agent-based simulation is very well suited for this purpose, as it allows to examine the effect of heterogeneity in socio-economic characteristics, and in isolation from other market properties that may affect ridesourcing performance. Such conditions may include the quality of alternative modes of transportation, ridesourcing pricing and imposed regulations.

Previous agent-based modelling exercises have provided insights into ridesourcing as a first / last mile solution for public transport^28,29^, driver earnings^25,28,30^, platform pricing policies^25,28,30,31^, the effect of labour market characteristics^25,28,30^, ride-pooling system performance^32–34^, and the design of charging infrastructure for an electric fleet^35,36^. An agent-based representation of the ridesourcing market was also used for studying income inequalities resulting from ridesourcing operations^19^. In this study, in contrast, we investigate the dependency of ridesourcing platforms on socio-economic inequalities, including pricing decisions of profit-maximising platforms, which has hitherto remained unexplored. Due to network effects in ridesourcing, selecting a profit-maximising strategy entails a complex trade-off between market volume and the profit per transaction^25^, of which the outcome is not obvious. At the same time, it is not evident how socio-economic inequality affects the distribution of social welfare between travellers and workers, which depends on the ratio between supply and demand^25^.

We fill this knowledge gap by adopting an agent-based simulation model of double-sided ridesourcing markets, MaaSSim^37^, integrated into a day-to-day framework representing network effects in the ridesourcing market^25^. The model simulates daily labour supply decisions in consideration of the anticipated income and the reservation wage of ridesourcing, i.e. the minimum wage required to be willing to work for the platform. The latter depends on a job seeker’s access to alternative employment opportunities. Mode choice is represented with a logit model incorporating several attributes, including the anticipated waiting time when opting for ridesourcing. Platform earnings and waiting time are learned primarily from personal experience, determined with an operational ride-hailing model in which travellers and drivers are matched based on their proximity^37^. The adopted simulation model also accounts for diffusion of platform awareness which may hinder market participation in early phases. In addition, it captures suppliers’ (medium- to long-term) trade-offs between registration costs (which are negligible for travellers) and anticipated participation benefits.

As participation is modeled endogenously on both sides of the market (as daily labour supply and mode choice), the day-to-day ridesourcing model generates disaggregate output on which travellers and job seekers participate in the market, depending on their socio-economic properties. This is in turn used to identify how socio-economic inequality affects ridesourcing system performance indicators, considering (exclusively) the direct effect of heterogeneity in travellers’ and job seekers’ time perceptions. This includes analysing how a profit-maximising platform adjusts its pricing strategy depending on the distribution of income in society, evaluating also the implications for riders and drivers.

## Application and results

We apply the double-sided ridesourcing market simulation model to a case study resembling the city of Amsterdam, the Netherlands. In addition to ridesourcing, travellers can choose between private car, bike and public transport. The first three modes operate on a road network with the same spatial configuration as the road network of Amsterdam, yet with universal (mode-specific) link travel speeds. Public transport service quality is based on GTFS data for Amsterdam.

Each traveller and each job seeker in the simulation represents 10 individuals in reality, in correspondence with other transportation studies in which agent-based models are applied^25,38,39^. This procedure results in 75,000 traveller agents and 2,500 job seeker agents that can potentially participate in the ridesourcing market in Amsterdam. Trip attributes are taken from data generated based on the activity-based model of Albatross^40^. Behavioural preferences related to travel and employment choices are specified based on findings reported in past studies^41–46^.

Model outputs include the profit generated by the platform, the share of requests that are satisfied, the average time travellers spent waiting before being picked-up, and the income of drivers. In addition, we formulate subsequent surplus-based welfare indicators for travellers and drivers.

We investigate the effect of socio-economic inequalities by adjusting the parameter $\sigma$ of the log-normal distributions used to describe heterogeneity in travellers’ value of time (VoT) and job seekers’ ridesourcing reservation wage. Our experiments cover scenarios for the entire spectrum ranging from perfect equality to extreme inequality, i.e. standard deviations resulting in Gini coefficients *g* between 0 and 0.95 (in steps of 0.05). In the following, we focus primarily on scenarios corresponding to urban Gini coefficients observed in the real world. This concerns Gini coefficients (in terms of disposable income) between 0.23 (Astana) and 0.67 (Curitiba, Pretoria and Johannesburg) ^47^. Most European and US cities fall in the middle of this range (e.g. Oslo 0.27, Amsterdam 0.37 and (Greater) New York 0.42). We devote a separate subsection to discuss ridesourcing system performance under extreme (i.e. unobserved) (in)equality.

### Macroscopic effects

**Figure 2:**
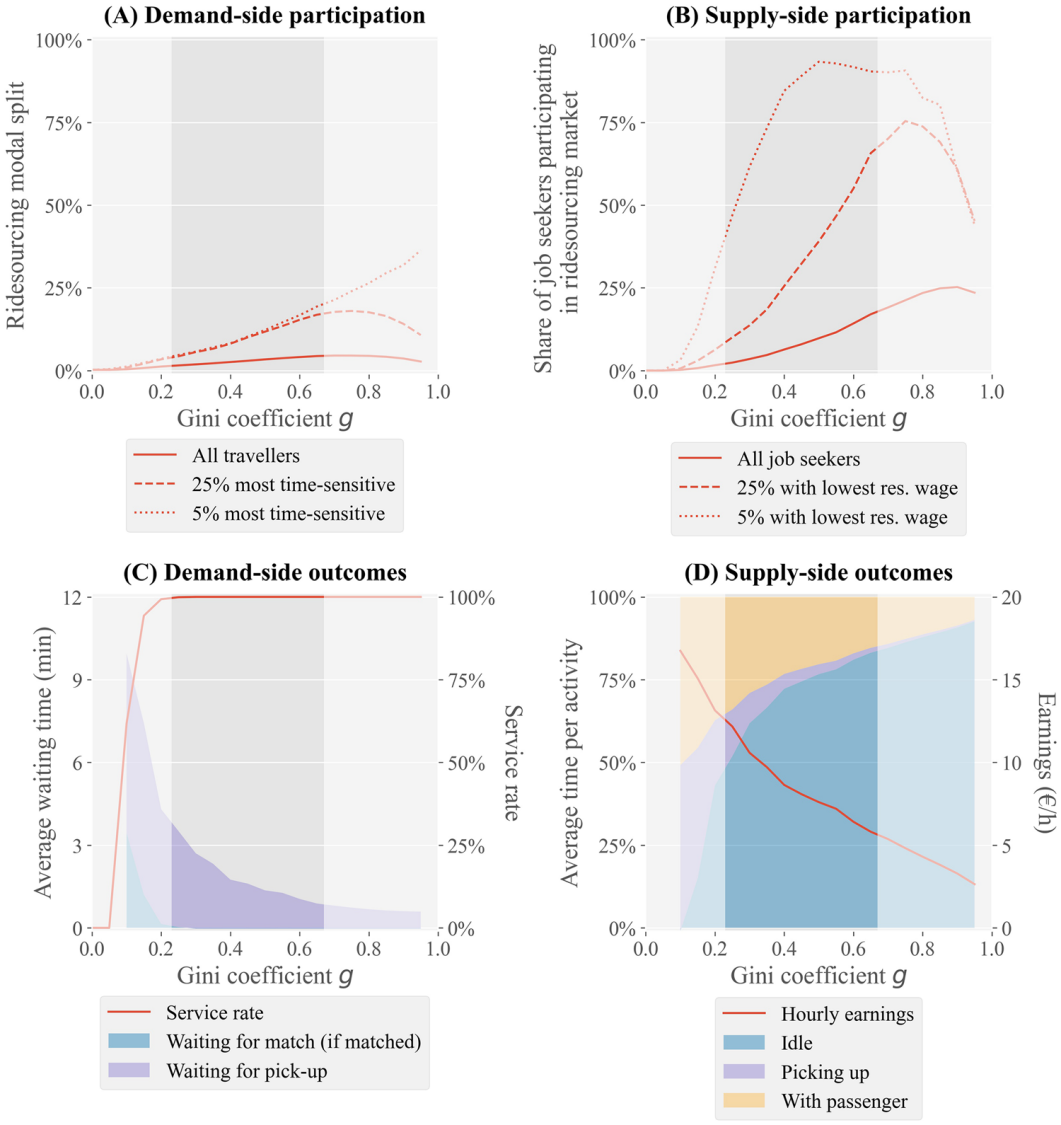
Key ridesourcing system indicators as a function of the degree of socio-economic inequality in society. (**A**) Modal split of ridesourcing, for the entire population as well as for the 25% and 5% most time-sensitive travellers (i.e. those with the highest willingness to pay for travel time reductions). (**B**) Share of job seekers participating in the ridesourcing market, for the entire population as well as for the 25% and 5% of job seekers with the lowest reservation wage. (**C**) Level of service indicators: service rate (share of requests that is satisfied) and customer waiting time before pick-up. (**D**) Driver activities and income. The highlighted area in each graph corresponds to observed values of the Gini coefficient in cities around the world.

Our experiments show that within the range of observed urban Gini coefficients, the greater the socio-economic inequality is, the higher the participation on both sides of the ridesourcing market is. Figure 2A suggests a strong near-linear positive relationship between socio-economic inequality and demand for ridesourcing. To illustrate, ceteris paribus, a ridesourcing market attracts approximately three times as much travel demand in an area with a Gini coefficient of 0.65 compared to one in an area with a Gini of 0.25 (4.4% vs. 1.5% market share). The relative increase in market participation is even larger on the supply side of the market. Here, we observe an exponential increase in market participation with growing socio-economic inequality (Fig. 2B). When *g* is 0.65, on average 17.0% of all job seekers work in the ridesourcing market, compared to just 2.4% when $g=0.25$.

**Figure 3:**
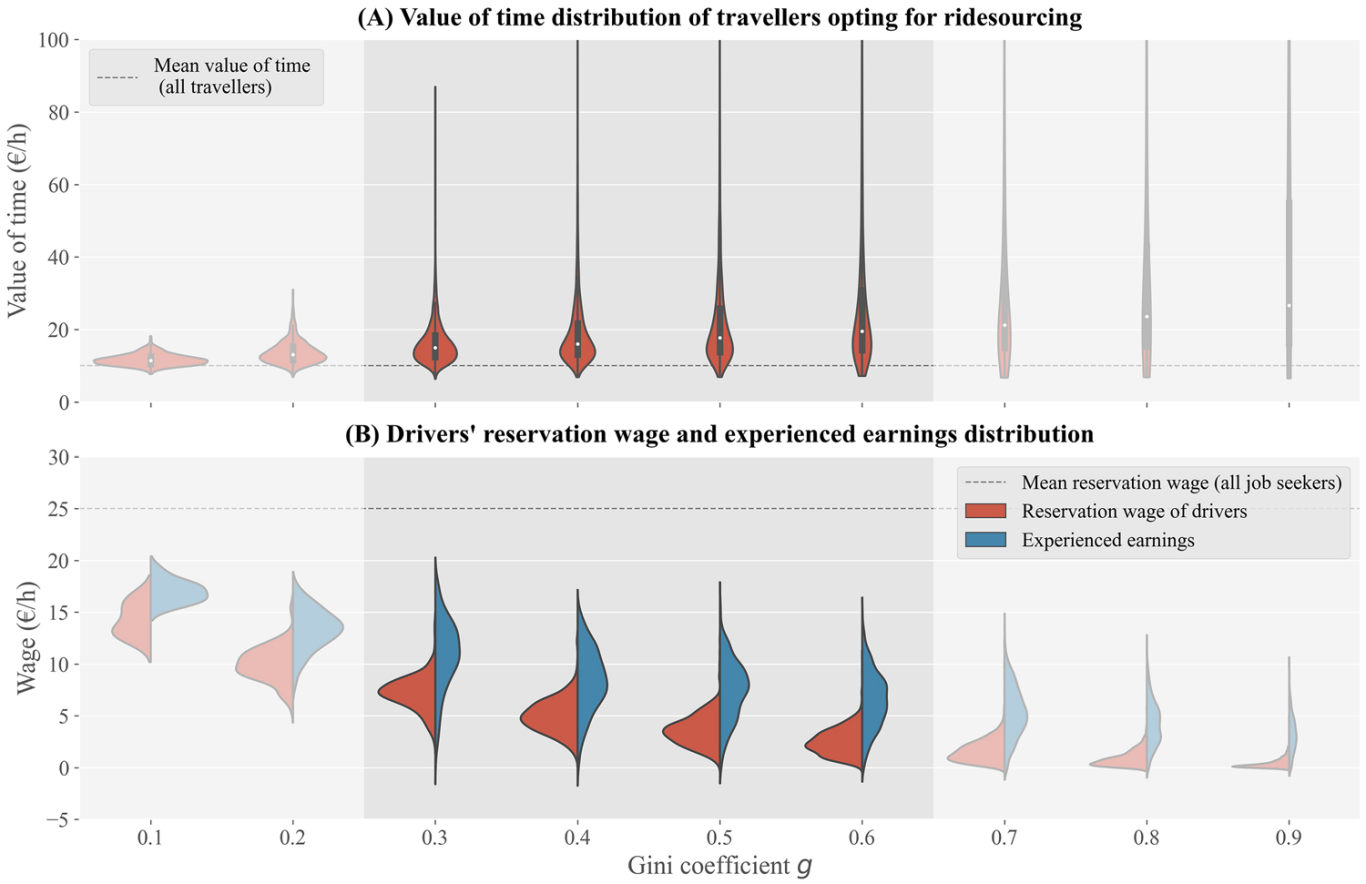
Distribution of (**A**) value of time of travellers opting for ridesourcing, and (**B**) reservation wage and earnings of job seekers participating in the ridesourcing market, for different values of Gini coefficient *g*. Mean value of time and reservation wage are provided for the entire population as reference. The highlighted area in each graph corresponds to observed values of the Gini coefficient in cities around the world.

**Figure 4:**
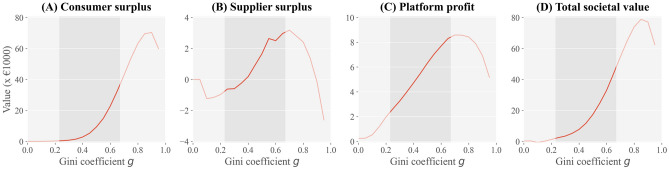
Social welfare generated by ridesourcing markets depending on the degree of socio-economic inequality. We present four indicators^25^. (**A**) Consumer surplus, defined as the increase in logsums when ridesourcing is added to the choice set. (**B**) Supplier surplus, defined as the summed difference between job seekers’ earnings and costs associated with the ridesourcing market, including labour opportunity costs and registration costs. (**C**) Profit from commissions. (**D**) The total social welfare, defined as the sum of the previous three. The highlighted area in each graph corresponds to observed values of the Gini coefficient in cities around the world.

The difference in income inequality elasticities between supply and demand sides of the market implies that ridesourcing markets operating in more unequal societies tend to end up in a more oversupplied market state. When comparing an environment where $g=0.25$ to one where $g=0.65$, approximating the most equal and the most unequal urban region worldwide, respectively, the ratio of supply to demand more than doubles. While in both scenarios customers are guaranteed instant assignment to a driver (Fig. 2C), the average distance between matched travellers and drivers decreases with socio-economic inequality, particularly in the lower range of social inequality. This results in shorter waiting times for travellers (down to less than a minute between requesting a ride and the assigned driver’s arrival at the pick-up location when $g=0.65$, Fig. 2C) and less deadheading for drivers (Fig. 2D). Yet, due to strong competition for rides, drivers spend substantially more of their time in an idle state (i.e. waiting to be matched): 83% vs. 52% (Fig. 2D). Consequently, driver earnings are, ceteris paribus, considerably lower in contexts where socio-economic inequality is high (Fig. 2D). In our experiment, the average driver earns €5.81 per hour when $g=0.65$ compared to €12.17 when $g=0.25$.

### Societal implications

Our results demonstrate that the extent to which ridesourcing is more popular among travellers with an above average value of time depends on the socio-economic inequality level (Fig. 3A). For instance, when *g* equals 0.25, of the 5% travellers with the highest value of time, 4.7% chooses ridesourcing as their mode of travel for their trip (Fig. 2A), around three times the population average. When *g* is 0.65, almost 1 in 5 individuals in the respective group opts for ridesourcing, nearly five times the population average. The ridesourcing market then attracts travellers with an average value of time of more than 20 euro per hour, including some extremely time-sensitive travellers, compared to an average value of time of approximately 13 euro per hour when $g=0.25$.

We observe even greater differences in supply-side participation depending on job seekers’ reservation wage. When $g=0.25$, of the 5% individuals with the lowest reservation wage, 46.9% participate in the ridesourcing market on an average day (Fig. 2B), compared to just 2.9% for the entire population of job seekers. This results in an average driver reservation wage of just over a third of the average job seekers’ reservation wage. The participation rate of the 5% job seekers with the lowest reservation wage peaks at $g=0.5$ (93.3%), after which it marginally decreases due to diminishing earnings. Yet, if we take the 25% job seekers with the lowest reservation wage instead, we find increasing participation rates beyond $g=0.5$ (Fig. 2B). When *g* exceeds 0.6, the average ridesourcing driver’s reservation wage lies below €2 per hour (Fig. 3B), less than 10% of the population average. Fig. 3B demonstrates that the average hourly ridesourcing earnings exceed the average reservation wage of drivers by €2-€4 independent of the geographical area’s Gini coefficient. Since both indicators vary between drivers, particularly earnings, not all drivers earn more than their reservation wage.

We observe that the consumer surplus increases exponentially with the Gini coefficient (Fig. 4A). This increase stems from (i) ridesourcing becoming more attractive due to increased supply, and (ii) the presence of highly time-sensitive travellers that benefit considerably from a direct service. Fig. 4B shows that in scenarios with limited inequality, the aggregated supplier surplus, which also accounts for costs associated with registration (the ability to participate), may be negative. Due to the relatively low participation probability in this case, registration costs are not offset by participation earnings. Platform profit increases linearly with the Gini coefficient (Fig. 4C). Considering fixed pricing, this follows directly from a linear increase in ridesourcing demand (Fig. 2A). In socio-economically unequal urban areas, the societal value generated by ridesourcing platforms, defined as the sum of the consumer surplus, supplier surplus and platform profit, consists predominantly of the consumer surplus (Fig. 4D).

### Pricing

**Figure 5:**
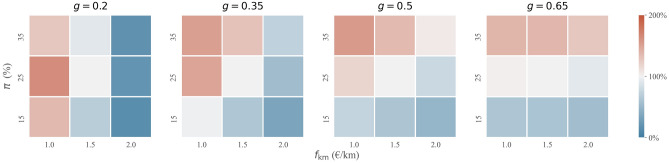
Platform profit depending on platform’s pricing strategy and the degree of socio-economic inequality in society. Profit is given relative to the profit under reference strategy with $f_{{{\rm km}}}=1.5$ €/km and $\pi =25\%$.

**Figure 6:**
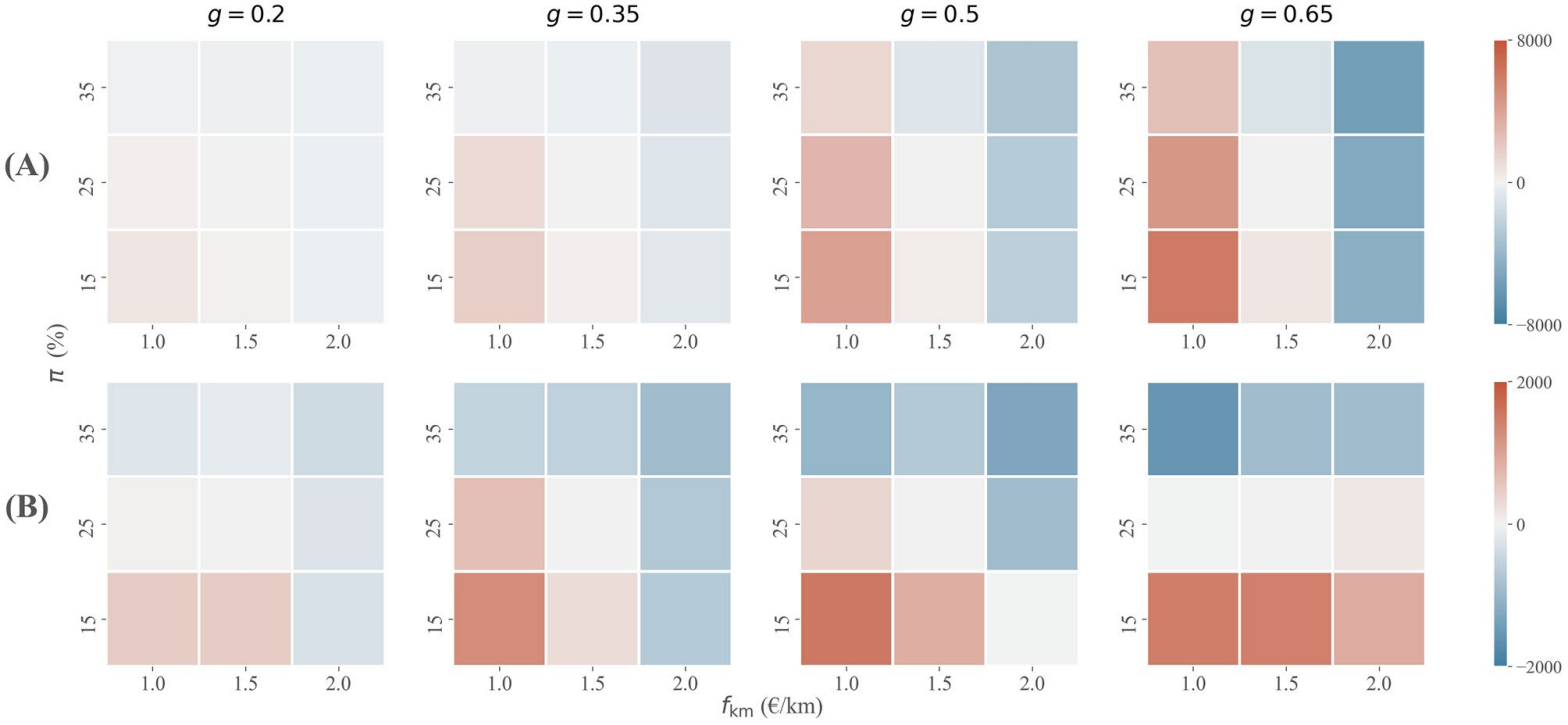
(**A**) consumer surplus and (**B**) supplier surplus depending on platform’s pricing strategy, for different levels of socio-economic inequality. Values are relative to reference pricing strategy with $f_{{{\rm km}}}=1.5$ €/km and $\pi =25\%$.

In all previous scenarios, the platform per-kilometre fare $f_{{{\rm km}}}$ and the commission rate $\pi$ were set to €1.5 and 25%, respectively, based on Uber’s pricing in Amsterdam^48^. We hypothesize, however, that a higher degree of socio-economic inequality allows for higher fares - as the demand-side target group of ridesourcing becomes more cost-insensitive - and a higher commission rate - as the supply-side target group of ridesourcing becomes more insensitive to income. We therefore proceed with analysing platform profit and other social welfare indicators for eight alternative pricing strategies, with $f_{{{\text{km}}}} = \left\{ {{\euro} 1,{\euro} 1.5,{\euro} 2} \right\}$ and $\pi =\{15\%,25\%,35\%\}$. Furthermore, we do so for four different levels of socio-economic inequality, i.e. $g=\{0.2,0.35,0.5,0.65\}$. The results are presented in Figs. 5 and 6.

We find that when $g=0.2$, a low per-kilometre fare is crucial to maximising platform profit (Fig. 5). A platform then generates 63.4% more profit when opting for a per-kilometre fare of €1 instead of €1.5, assuming an optimal 25% commission rate. This strategy yields a nearly 50% increase in demand-side participation and a 15% increase in supply-side participation. The moderate commission rate of 25% is then found optimal for the platform whereas a high commission rate, in combination with low fares, deters too many job seekers. In contrast, a low commission rate generates insufficient induced demand (through reduced waiting following from latent supply) to compensate for a lower profit per satisfied request.

When $g=0.65$, we find that the per-kilometre fare $f_{{{\rm km}}}$ has a limited effect on platform profit. The reason is that induced demand from lower fares (while accounting for changes in supply) is counteracted by a lower platform profit per market transaction. Conversely, the commission rate $\pi$ is a crucial platform pricing instrument for profit maximisation in socio-economically unequal societies. A commission rate of 35% compared to 25% (with $f_{{{\text{km}}}} = {\euro} 1.5$) for instance yields 36.9% more platform profit. Having a very low reservation wage, few drivers are deterred when a platform opts for such a commission (14.8% of drivers), so that the increase in platform commission only results in a marginal reduction of ridesourcing demand (2.3%). In terms of platform profit, the lower market share is substantially compensated for by a higher profit margin on remaining demand.

Platform pricing decisions may not only strengthen the previously proposed (positive) relationship between socio-economic inequality and platform profit (in Fig. 4C), but also the (negative) relationship between socio-economic inequality and driver earnings (in Fig. 2B). Figure 6B shows that a higher commission rate results in a substantially lower supplier surplus, suggesting that profit-maximising behaviour in socio-economically unequal societies is likely to come at the expense of drivers. Such a strategy also harms travellers, albeit to a much lesser extent as the relation between commission and consumer surplus is much less pronounced (Fig. 6A). In areas with limited inequality, on the other hand, profit-maximising strategies are less costly for participants in the two-sided market. Due to the presence of few job seekers with a low reservation wage, the platform is more restricted in setting its commission. In fact, in a relatively equal society a profit-maximising pricing strategy entails charging low fares, which is likely to benefit travellers at limited to no cost for drivers (the lower revenue per satisfied trip request is offset by reduced idle time through induced demand).

### Extreme (in)equality

In the preceding, we provided evidence for a positive relationship between socio-economic inequality and ridesourcing market share within the range of observed inequality. In this subsection, we analyse ridesourcing performance under degrees of socio-economic inequality which extent beyond the range of those currently observed amongst urban societies worldwide.

First, we observe that in hypothetical societies with *g* above the observed maximum of 0.67, (a further) increase in Gini coefficient does not induce a further increase in (demand-side) ridesourcing market share (Fig. 2A). In fact, from $g=0.8$ onward, there is a strong negative relationship between inequality and ridesourcing modal split, and thereby platform profit. The reason is that, in such societies, few travellers have an extremely high value of time, while everyone else is very insensitive to travel time and therefore unlikely to opt for the relatively expensive ridesourcing service. With few customers and many drivers willing to work for little, driver earnings are very low in highly unequal societies, down to less than €3 per hour on average when $g=0.95$ (Fig. 2D). Travellers, on the other hand, experience a supreme service with just a 1 min average pick-up time (Fig. 2C).

At the other end of the spectrum, i.e. for *g* below the minimum observed real-world value of 0.23, we observe that the ridesourcing market ends up in an undersupplied state. Even though drivers face little to no matching time given limited competition for requests, their earnings are limited by considerable deadheading for picking up customers (Fig. 2D). With very little heterogeneity in reservation wages, few job seekers are willing to drive for the resulting wage. At the same time, the ridesourcing platform fails to attract substantial demand, which is required to minimise drivers’ deadheading, because (i) there are few time-sensitive travellers that otherwise benefit most from the existence of ridesourcing, and (ii) the presence of few drivers implies a poor level of service. In other words, ridesourcing dependency on network effects - i.e. few market participants resulting in low matching efficiency - can exacerbate general unwillingness to participate in the market when the pools of travellers and drivers are relatively homogeneous in terms of their socio-economic properties. This indicates that operating a ridesourcing platform in a society with limited socio-economic inequalities may not be viable. In fact, in our experiment when *g* is below 0.1, not a single job seeker is willing to drive for the platform (Fig. 2B).

## Discussion

Our findings show that the profit generated by ridesourcing platforms increases substantially with socio-economic inequality. We identify four (intertwined) mechanisms which contribute to this relation. First, there are more time-sensitive travellers in unequal societies. These travellers are relatively likely to opt for the direct, private service offered by ridesourcing platforms. Second, there are more job seekers willing to participate for limited market earnings in such societies. In fact, we observe that ridesourcing supply is even more elastic than demand in relation to inequality, so that ridesourcing markets are more likely to end up substantially oversupplied in unequal societies. This results in an improved level of service due to shorter waiting times. The difference between supply and demand elasticity (in relation to inequality) arguably stems from two underlying reasons: (i) asymmetry in the distributions of reservation wage and value of time, i.e. both distributions are right-skewed, implying that the majority of individuals have a below average value of time / reservation wage, and (ii) that income is likely more important in working decisions than travel time is in mode choice; whereas at the same time it is undeterred by decreasing driver earnings and increased level of service as inequality grows. Third, increased market participation (on both sides) yields better quality matches, i.e. the average pick-up distance decreases with the scale of the market. This results in an even better level of service, and ultimately, a larger market share. Fourth, the abundant presence of job seekers with a low ridesourcing reservation wage allows the platform to raise its commission rate, which yields a higher profit per served traveller.

Whereas socio-economic inequality is beneficial for companies operating ridesourcing markets as well as for travellers requesting rides on these platforms, the earnings of drivers participating in these markets decrease considerably with socio-economic inequality. This is the outcome of increased competition for ridesourcing requests as a result of the abundance of job seekers with a low ridesourcing reservation wage in socio-economically unequal societies. Furthermore, this may be aggravated by profit-maximising pricing decisions, i.e. platforms opting for a higher commission under large inequality. Based on our experiments, it is highly unlikely that ridesourcing markets evolve towards an undersupplied market equilibrium, a market state potentially benefiting drivers. In our experiments, it only occurs for Gini coefficient *g* of 0.15 or smaller, whereas the minimum value observed in reality is 0.23.

Our results indicate that societies with a high degree of socio-economic inequality are likely target markets for ridesourcing service providers. We observe low driver earnings particularly in these markets, down to less than one fifth of the average job seeker’s reservation wage of €25 per hour. Authorities may therefore consider policies aimed at improving the earnings of ridesourcing drivers in socio-economically unequal urban areas. This may include regulation of the commission, which we find to benefit travellers as well. Alternatively, driver earnings may be boosted with supply caps, as implemented for instance in New York City, aiming to reduce supply-side competition.

The presented results follow from applying an agent-based model representing the interactions between the key actors in the ridesourcing market to a case study resembling the city of Amsterdam. While this approach allows to explore the effect of the entire range of inequality levels—from perfect equality to extreme inequality—as well as platform’s pricing strategy in isolation from other factors, results should not be interpreted without caveats. For instance, our method assumes that travellers’ value of time and job seekers’ reservation wage follow a log-normal distribution (resulting in a similar Gini coefficient), based on an estimation of the distribution of (gross) income in the Netherlands. While there is evidence that the reservation wage of a job is related to income in previous work^49^, it is also influenced by other factors, including the (personal) perceived attractiveness of the job. Yet, given its association with income, it seems likely that the reservation wage distribution is in reality right-skewed, a key condition for the supply elasticity related to socio-economic inequality to exceed the corresponding demand elasticity. Furthermore, future research can account for spatial correlations in travellers’ and job seekers’ time perceptions associated with socio-economic inequality, i.e. spatial clustering of high and low income households, the effect of which on ridesourcing systems is uncertain. At the same time, heterogeneity in travellers’ and job seekers’ time valuations may not be the sole mechanism through which socio-economic inequality can affect ridesourcing systems. For instance, socio-economic inequalities may contribute to safety concerns^50–52^. In the literature, there is no consensus whether ridesourcing is perceived positively or negatively with regard to safety^53–55^, and therefore it is undetermined how such a consideration would affect the market share of ridesourcing.

All in all, we consider it probable that ridesourcing platforms thrive on socio-economic inequality. While the transportation and demand features vary from one location to another, the mechanism of cheap labour and time-sensitive ridesourcing users allowing for network effects and higher platform commission in unequal urban areas is likely universal. We recommend future research investigating exactly how context attributes such as labour market conditions, demand and transportation system features affect the relationship between socio-economic inequality and ridesourcing performance. We anticipate that repositioning - not captured in this work - may further contribute to a negative relationship between socio-economic inequality and ridesourcing drivers’ income. The reason is that travellers’ waiting times are already minimal (and the service rate maximal) without drivers repositioning, implying that repositioning - while potentially benefiting individual drivers in the short run - is likely to yield minimal induced demand at the system level, instead resulting only in additional operational costs for drivers.

It would be valuable to delve deeper into the intricate, bidirectional relationship between ridesourcing platforms and socio-economic inequality, in addition to examining the effect of other factors than inequality, such as the average income in the population. While a previous study has touched upon how ridesourcing platforms may contribute to socio-economic inequality^19^, their analysis omitted the daily decisions made by both ridesourcing users and job seekers. This omission presents an opportunity to investigate how these decisions influence the dynamics of inequality. Understanding the potential existence and magnitude of a reinforcing feedback loop in this context could shed light on the complexities of the ridesourcing market’s influence on socio-economic disparity, and vice versa.

Finally, examining the impact of socio-economic inequalities on various mobility services beyond ridesourcing could also offer intriguing insights. At the same time, we expect that gig platforms outside of passenger mobility may benefit in a similar way from socio-economic inequality as ridesourcing providers do. This applies particularly to markets for low-skill services that are most interesting for cost-insensitive individuals, such as platforms for household jobs, meal delivery or grocery delivery.

## Methods

### Travel demand data

Travel demand is taken from a data set generated with an activity-based model for the Netherlands^40^, selecting trips with origin and destination in the studied area, with a trip distance of at least 2 kilometres, and a request time between 09:00 and 18:00. Travellers’ value of time is drawn from a log-normal distribution with a mean of €10 per hour, and a standard deviation corresponding to Gini coefficient *g*. Each day, travellers make the same trip, for which they reconsider their travel mode on a daily basis. To reduce the computational complexity of the simulation, all travellers with a below 5% probability of choosing ridesourcing when no waiting time is anticipated are assumed to never opt for ridesourcing.

### Modelling framework

**Figure 7:**
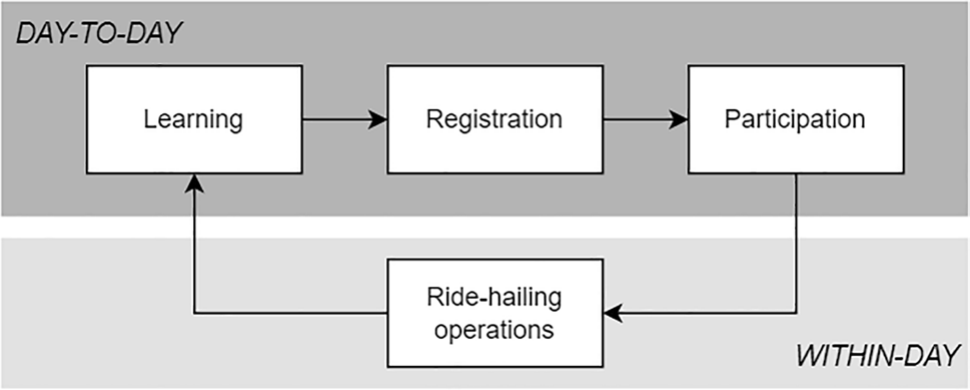
Day-to-day processes captured in the simulation model, for a single side of the ridesourcing market.

Our model captures daily participation decisions in the ridesourcing market (Fig. 7). We thereby represent how agents learn from individual participation experience, or through the experience gained by others. Experiences are represented with a within-day operational model for ride-hailing^37^, based on double-sided participation volumes. Our day-to-day model accounts for barriers to participation, i.e. platform registration. This requires platform awareness and trading off potential investments associated with the ability to participate in the market against anticipated participation earnings. In the ensuing we describe the processes captured in our model in more detail.

#### Participation

Assume job seeker *s* has a reservation wage $r_s$, drawn once from a log-normal distribution with a mean of €25 per hour and a standard deviation that yields Gini coefficient *g*. It represents the minimum wage for which they are - on a typical day - willing to drive for the ridesourcing platform. With this assumption, our model follows the neoclassical theory of labour supply^56–58^. Our participation model considers that variables other than the anticipated income $\hat{i}_{st}$ (for day *t*) and the reservation wage $r_s$ may play a role in participation decisions, by means of applying a random utility model with sensitivity parameter $\beta _{{{\rm ptp}}}$ and error term $\varepsilon _{{{\rm ptp}}}$. The utility and the resulting probability that job seeker *s* participates in the market on day *t* are as follows:

1 $$U_{{st}}^{{{\text{participate}}}} = \beta _{{{\text{ptp}}}} \cdot (\hat{i}_{{st}} - r_{s} ) + \varepsilon _{{{\text{ptp}}}} {\text{ }}$$

2 $$\begin{aligned} p^{{{\rm participate}}}_{st}&= \frac{1}{1+\exp (-U_{st}^{{{\rm participate}}})} \end{aligned}$$

At the same time, each day traveller *c* chooses a transport mode from a choice set *M*. In addition to ridesourcing, this set contains three alternative modes: private car, bike and public transport. Walking is not considered given its limited modal share for trips longer than 2 km^59^. Travellers consider five attributes in their mode choice: in-vehicle time $v_{cm}$, the sum of access and egress time $a_{cm}$, waiting time $\hat{w}_{ctm}$, the number of required transfers $q_{cm}$, and travel cost $\rho _{cm}$. The choice model parameters associated with these attributes are denoted $\beta _{m}^{{{\rm ivt}}}$, $\beta _{m}^{{{\rm access}}}$, $\beta _{m}^{{{\rm wait}}}$, $\beta _{{{\rm transfer}}}$, and $\beta _{{{\rm cost}}}$, respectively. Only the stop waiting time $\hat{w}_{ctm}$ of ridesourcing is dynamic, i.e. depending on supply and demand levels on that particular day. Mode-specific preferences are captured by means of an alternative-specific constant $ASC_{cm}$. A random utility model with an error term $\varepsilon _{{{\rm mode}}}$ is applied to account for unobserved variables in the mode choice. The utility associated with mode *m* and the probability of choosing that mode are as follows:

3 $$\begin{aligned} U_{ctm}^{{{\rm mode}}}&= \beta _{m}^{{{\rm ivt}}} \cdot v_{cm} + \beta _{m}^{{{\rm access}}} \cdot a_{cm} + \beta _{m}^{{{\rm wait}}} \cdot \hat{w}_{ctm} + \beta _{{{\rm transfer}}} \cdot q_{cm} + \beta _{{{\rm cost}}} \cdot \rho _{cm} + ASC_{cm} + \varepsilon _{{{\rm mode}}} \end{aligned}$$

4 $$\begin{aligned} p^{{{\rm mode}}}_{ctm}&= \frac{\exp (U_{ctm}^{{{\rm mode}}})}{\sum _{m\in M} \exp (U_{ctm}^{{{\rm mode}}})} \end{aligned}$$

#### Ride-hailing operations

We adopt an agent-based model, *MaaSSim*, for simulating the within-day dynamics of ride-hailing systems^37^, in order to obtain passengers’ waiting time and drivers’ income depending on supply and demand levels. Each day, 8 h are simulated. Participating drivers work all 8 h, i.e. there is no work shift choice. We assume that drivers lease their vehicles, bearing per-kilometre operational costs of 0.25 €/km^60^, which covers for fuel, insurance, and minor maintenance costs. Matching takes place whenever there is a non-empty virtual queue of unassigned requests and a non-empty virtual queue of idle drivers. The platform agent assigns idle drivers to pending requests based on their proximity, i.e. the closest pair is selected. Travellers and drivers accept all match offers. A ride request is revoked if unanswered after 10 min, which is perceived as 30 min of waiting to account for inconvenience associated with not finding a driver. Drivers remain idle at their last drop-off location until assigned to a new request. When driving, they move at a (constant) speed of 36 km/h^61^. The platform applies a distance-independent base fare $f_{{{\rm base}}}$, an additional per-kilometre fare $f_{{{\rm km}}}$, and a commission rate $\pi$.

#### Learning

We apply a Markov model to represent learning from experience, i.e. each agent assigns a weight of 20% to their last experience as opposed to all previous information. Agents that are not registered do not gain first-hand experience and instead rely on information from other (registered) agents. Each day, each passenger (driver) receives a private signal of the waiting time (earnings) in the system, which we draw from a random distribution with mean equal to the average experienced waiting time (earnings), and standard deviation equal to 0.5 times the standard deviation of the distribution of experienced waiting time (income).

#### Registration

We assume that agents can only participate in the market if they have been informed about the existence of the service. We model this with two (separate) epidemic compartment models, one for job seekers and one for travellers. The probability to be informed on day *t* depends on the share of fellow job seekers or travellers, respectively, that have been previously informed, and information transmission rate $\psi$.

In addition, we assume that driving for the platform—unlike requesting a ride on the platform-requires medium—to long-term investments, representing entering into vehicle leasing and insurance contracts for instance. Every day, (informed) job seekers have a 10% probability of evaluating their registration status. We assume that the daily costs associated with being registered (vehicle leasing costs) are 20 euros^62^, which cannot be cancelled in the first 5 days after registration. In (de)registration decisions, they consider participation probability $p^{{{\rm participate}}}_{st}$, i.e. the fact that participation earnings $\hat{i}_{st}$ may need to outweigh reservation wage $r_s$ by more than the daily registration costs, to cover for participation-independent registration costs on days that they do not participate in the market. (De)registration choice is—similar to participation choice—modeled with a random utility model, with sensitivity parameter $\beta _{{{\rm reg}}}$ and error term $\varepsilon _{{{\rm reg}}}$.

### Implementation

The simulation is terminated once both the average expected waiting time and the average expected income has changed by less than 1% for 5 days in a row. The model is implemented in Python. We replicate the experiment for statistical significance, with the number of replications based on the average anticipated driver earnings and the average anticipated user waiting time.

### Supplementary Information

Supplementary Information.

## Data Availability

Network data for Amsterdam is retrieved from OpenStreetMap, public transport itineraries from OpenTripPlanner. Travel demand is harvested from the Albatross data set ^40^. The code to generate ridesourcing system performance indicators in market equilibrium for given socio-economic inequality and pricing settings is available in the following public GitHub repository: https://github.com/Arjan-de-R/MaaSSim. This repository is a fork of the MaaSSim repository: https://github.com/RafalKucharskiPK/MaaSSim.
