# Supplementary material for: Ridesourcing platforms thrive on socio-economic inequality
Source: Sci Rep. 2024 Mar 28;14:7371. doi: 10.1038/s41598-024-57540-x (PMC10978858; doi:10.1038/s41598-024-57540-x)
Supplement: Supplementary file 1 — Supplementary Information. [file 41598_2024_57540_MOESM1_ESM.pdf]

Supplementary information for the paper *Ridesourcing Platforms Thrive on Socio-Economic Inequality*

Arjan de Ruijter[1,*], Oded Cats[1], and Hans van Lint[1]

[1]Delft University of Technology, Transport & Planning, Delft, 2628 CN, Netherlands
[*]a.j.f.deruijter@tudelft.nl

1 Gini coefficient of the lognormal distribution

The following formula is used to convert between lognormal parameter σ and Gini coefficient g:

$$\sigma = 2\,\mathrm{erf}^{-1}(g) \tag{1}$$

2 Replications

As our simulation model contains several processes with stochastic components (i.e. participation choice, registration choice and the diffusion of platform awareness), we replicate the experiment for statistical significance. To determine the number of required replications for each scenario, we apply a method originally used in traffic simulations [1, 2]. We denote I^* as the average anticipated ridesourcing income by (registered) job seekers in equilibrium in a single iteration, and W^* as the corresponding average anticipated waiting time of (informed) travellers. We define $\overline{I^*}(m)$ and $\overline{W^*}(m)$, and $s_{\mathrm{i}}(m)$ and $s_{\mathrm{w}}(m)$, respectively, as the estimated mean and standard deviation of I^* and W^*, based on a sample of m runs. We denote the allowable percentage error of estimate $\overline{I^*}(m)$ and $\overline{W^*}(m)$ compared to the actual mean as $\varepsilon_{\mathrm{repl}}$, and the level of significance as α. Then, the minimum number of replications based on a sample of m runs is:

$$Z(m) = \max\left(\left(\frac{s_{\mathrm{i}}(m)\cdot t_{m-1,\frac{1-\alpha}{2}}}{\overline{I^*}(m)\cdot\varepsilon_{\mathrm{repl}}}\right)^2, \left(\frac{s_{\mathrm{w}}(m)\cdot t_{m-1,\frac{1-\alpha}{2}}}{\overline{W^*}(m)\cdot\varepsilon_{\mathrm{repl}}}\right)^2\right) \tag{2}$$

3 Experimental set-up

3.1 Alternative modes

Private cars use the same road network as ridesourcing vehicles and operate at the same speed. Private car users require 10 minutes to access and park their vehicle, and face per-kilometre costs of 0.5 €/km [3], as well as (fixed) parking costs at their destination. These parking costs are 15 euro in the city centre (i.e. the area enclosed by IJ river and Singelgracht), and 7.5 euro elsewhere. Bikes operate on the same network, albeit with a 2.5 times lower speed [4]. The choice for a bike comes without costs or access / parking time. For public transport, travellers consider the itinerary with the earliest possible arrival time based on their trip request time, queried using OpenTripPlanner based on a representative weekday (November 1st, 2021). Public transport fares are based on the fare scheme operated by Amsterdam's public transport provider GVB on this same date.

3.2 Mode choice parameters

One minute of walking and waiting time are perceived 2 and 2.5 times more negatively than one minute of in-vehicle time [5], i.e. $\beta_m^{\text{access}} = 2 \cdot \beta_m^{\text{ivt}}$ and $\beta_m^{\text{wait}} = 2.5 \cdot \beta_m^{\text{ivt}}$. Each transfer in public transport is perceived as 5 minutes of in-vehicle time [6]. Bike time is perceived twice as negative as in-vehicle time [7, 8]. Cost parameter β_{cost} and alternative specific constants (ASCs) are taken from a study investigating urban travel in the Netherlands [9].

3.3 Other model parameters

Table 1 presents the specification of the remaining model parameters.

Table 1: Specification of model parameters.

**Parameter**	**Value**	**Unit**	**Description**
ψ	0.1	-	Information transmission speed
β_{reg}	0.2	util/€	Income sensitivity in registration
β_{ptp}	0.1	util/€	Income sensitivity in participation
$\varepsilon_{\text{repl}}$	0.1	-	Allowable percentage error of estimate of mean
α	0.05	-	Level of significance

3.4 Initialisation

Job seekers and travellers have a 10% probability to be aware about the platform at the start of the simulation. Informed job seekers have an initial 20% probability to be registered. Lacking experience, they expect earnings equal to their reservation wage. Informed travellers expect no waiting time.

References

[1] Kazi Iftekhar Ahmed. *Modeling drivers' acceleration and lane changing behavior.* PhD thesis, Massachusetts Institute of Technology, 1999.

[2] Wilco Burghout. A note on the number of replication runs in stochastic traffic simulation models. *Unpublished report, Stockholm: Centre for Traffic Research*, 2004.

[3] Nibud. Autokosten. https://www.nibud.nl/onderwerpen/uitgaven/autokosten, 2022. Accessed Sep 20, 2022.

[4] Fietstelweek. Resultaten fiets telweek 2016. https://fietstelweek.nl/resultaten-fiets-telweek-bekend/, 2016. Accessed Dec 16, 2022.

[5] Mark Wardman. Public transport values of time. *Transport policy*, 11(4):363–377, 2004.

[6] Menno Yap, Oded Cats, and Bart van Arem. Crowding valuation in urban tram and bus transportation based on smart card data. *Transportmetrica A: Transport Science*, 16(1):23–42, 2020.

[7] Maria Börjesson and Jonas Eliasson. The value of time and external benefits in bicycle appraisal. *Transportation Research Part A: policy and practice*, 46(4):673–683, 2012.

[8] Jeroen van Ginkel. The value of time and comfort in bicycle appraisal. Master's thesis, University of Twente, 2014.

[9] Nejc Geržinič, Niels van Oort, Sascha Hoogendoorn-Lanser, Oded Cats, and Serge Hoogendoorn. Potential of on-demand services for urban travel. *Transportation*, pages 1–33, 2022.
